# A Squib

**Published:** 1862-12

**Authors:** 


### A SQUIB.

THOSE who have been engaged in literature for any length of time, or who are familiar with the biographies of literary men, and, therefore, acquainted with the satires and lampoons which the best writers have been subjected to, soon learn to estimate such things at their proper value, and therefore either treat them with silent contempt, or amuse themselves and friends, as Dr. Johnson used to do, by showing how much better the thing might have been done.—*Exchange.*

Well, how many "satires and lampoons" does it take to make a "thing." We have tried a number of each on a friend; and he is not much of a *thing* yet. W.

